# Comparing Multigene Molecular Testing Results of MRI-Target Versus Systematic Prostate Needle Biopsies of Candidates for and Under Active Surveillance

**DOI:** 10.3390/jpm15070279

**Published:** 2025-07-01

**Authors:** Nicholas J. Lanzotti, Chris Du, Julia Hall, Joseph Saba, Maria M. Picken, Gopal N. Gupta

**Affiliations:** 1 Department of Urology, Loyola University Medical Center, Maywood, IL 60153, USA; 2 Stritch School of Medicine, Loyola University, Maywood, IL 60153, USA; 3 Department of Pathology, Loyola University Medical Center, Maywood, IL 60153, USA; 4 Department of Surgery, Loyola University Medical Center, Maywood, IL 60153, USA; 5 Department of Radiology, Loyola University Medical Center, Maywood, IL 60153, USA

**Keywords:** MRI, biopsy, prostate cancer

## Abstract

**Introduction:** The multigene molecular testing of prostate cancer tissue after biopsy provides individualized information to guide further management. The utility of selective genetic testing for MRI-visible target versus systematic cancer in patients as well as during different time points of active surveillance (AS) is unknown. The objective of this study was to compare Prolaris^TM^ results of MRI-target cancers versus systematic cancers on prostate needle biopsy as well as both during consideration for initial AS candidacy and candidacy for remaining on AS. **Methods:** Our prospectively maintained institutional multiparametric (mp) MRI prostate cancer active surveillance database (2013–2024) was queried for patients that underwent Prolaris^TM^ genetic testing of positive biopsy cores. Baseline information for PSA, PSA density, and Prolaris^TM^ calculated data were collected. Information on the timing of the Prolaris testing, defined as during the initial cancer diagnostic biopsy or on a subsequent confirmatory biopsy was collected. SPSS v29.0 was used to compare the selective Prolaris^TM^ results of MRI-target cancers versus systematic cancers during different points of AS. **Results:** 264 patients with a Prolaris^TM^ test were identified, 86 with MRI-target and 178 on systematic cancers. 182 Prolaris^TM^ tests were sent on a diagnostic biopsy and 81 on a subsequent biopsy. Overall, MRI-target cancers had similar risk scores (3.23 vs. 3.14, *p =* 0.18). Prolaris^TM^ scores were higher for GG2 systematic than GG1 target cancers (3.40 vs. 3.18, *p =* 0.023). The GG2 systematic lesion cohort also had higher predicted the 10-year disease-specific mortality (DSM) (3.40% vs. 2.30%, *p <* 0.01) and 10-year metastasis risk (1.90% vs. 1.20%, *p =* 0.013), and more aggressive recommended treatment. Analyses of the Prolaris^TM^ results sent during a diagnostic biopsy yielded similar results. Finally, on an analysis of the Prolaris^TM^ results sent during subsequent biopsy, a systematic GG2 biopsy was noted to have a higher 10-year DSM and metastasis rate, but similar risk scores and treatment recommendations. **Conclusions:** Prolaris^TM^ tests can be sent at multiple time points of AS, and selectively for MRI-visible versus higher grade cancers. There is no consistent association between MRI-visible cancer and Prolaris risk profile. When utilizing multigene molecular testing in prostate cancer, each individual patient must be evaluated to decide the appropriate level of care.

## 1. Introduction

Globally, prostate cancer is the second most common and fifth leading cancer specific cause of death in men [1]. Every year, over 1 new million cases of prostate cancer are diagnosed [2]. However, approximately 1 in 5 new diagnoses are low-risk prostate cancers, with a low risk of metastasis and cancer specific mortality [3,4]. As a result, aggressive treatment such as radical prostatectomy or radiation therapy are generally not recommended due to treatment associated comorbidities [5]. Active surveillance (AS), a means of monitoring men with less aggressive prostate cancer, is now the predominant management method for this group of patients [6,7]. Furthermore, for certain intermediate risk prostate cancers, AS has been shown to have excellent outcomes [8]. As a result, the American Urological Association (AUA) guidelines now recommend active surveillance as the preferred management option for low-risk and certain favorable intermediate risk prostate cancers [9].

Nevertheless, a great deal of clinical nuance in choosing between starting or continuing AS and moving to more definitive therapy for patients with high-volume low-risk prostate cancer and low-volume favorable intermediate-risk disease exists [10]. Adjunct modalities may provide additional information in these scenarios. Multiparametric MRI (mpMRI) has been shown to improve the detection of clinically significant Grade Group (GG) ≥2 prostate cancer and is recommended by the AUA guidelines [11,12]. However, an estimated 10–15% of clinically significant prostate cancers are not detected on MRI, leading to a continued need for systematic prostate biopsy tissue [13].

Tissue-based genomic biomarkers are an additional adjunct modality that can be used in a selective manner to help determine the next step for patients with equivocal biopsy and clinical findings [9]. The Prolaris^TM^ test, a post-biopsy adjunct, predicts likelihood of cancer-specific mortality via mRNA expression of cell cycle progression genes. Each increase by one point in score represents a doubling of prostate cancer-specific mortality [14,15]. It may be sent post-biopsy on a cancerous core to aid in decision making. The National Comprehensive Cancer Network guidelines states that these tests such as Prolaris^TM^ can be used for risk stratification after a diagnostic biopsy to identify patients for AS [16].

The heterogeneity of prostate cancer within the gland though, is an important consideration when sending a specimen for genetic testing. Typically, the most aggressive portion of the malignancy drives the patient’s risk profile [17,18]. Prior research has shown that MRI-visible prostate cancer patients may have more deleterious pathologies than MRI-invisible prostate cancer, and thus are less appropriate for AS [19,20,21]. However, the literature is limited on whether Prolaris^TM^-identified genomic risk characteristics are congruent with the deleterious characteristics of MRI-visible cancers.

**Figure 1:**
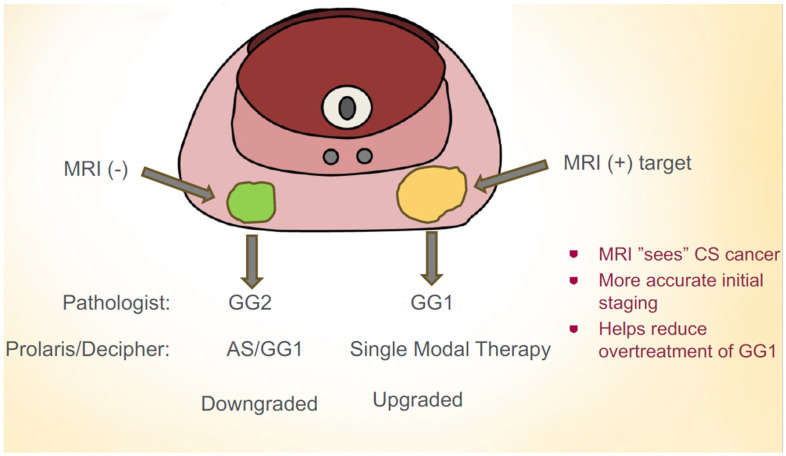
Hypothesis that MRI-target GG1 cancers may have worse prognosis than systematic GG2 cancers. A multigenetic molecular test may help guide management decisions in this scenario.

The impact of sending a Prolaris^TM^ test on an MRI-target versus a systematic cancer may lead to changes in one’s clinical decision making. Furthermore, during AS, approximately 10–15% patients will experience tumor progression on a subsequent biopsy [22]. The impact of sending a Prolaris^TM^ test on subsequent biopsies for patients on AS is unknown. The objective of this study was to compare Prolaris^TM^ tests for MRI-target compared to systematic cancers at two different time points of patients on AS: during a diagnostic biopsy and on a subsequent biopsy. We hypothesized that MRI-target cancers would have a higher genomic risk profile and that GG1 MRI-target cancers would have a more similar genomic risk profile as GG2 systematic cancers (Figure 1).

## 2. Methods

This study was a retrospective review of our Institutional Review Board (IRB)-approved (IRB LU 215920) Loyola University Medical Center prostate cancer active surveillance database from 2013 to 2024, which was deemed exempt given its retrospective nature. The database was queried for patients who had Prolaris^TM^ testing sent on their positive prostate biopsy. Patients must be candidates for active surveillance to be included in this study. The pre-biopsy age, PSA, and PSA density were recorded, as were the mpMRI relevant findings such as the presence or absence of an MRI-target and the PIRADS score(s) of the lesion(s). Both the systematic and the target lesion Gleason scores of the specific specimen core sent off for genomic analysis were recorded. The core sent off was the highest Gleason score within the specimen regardless of a target or systematic biopsy origin to evaluate the patient’s initial or continued candidacy for AS. All available information from the genetic test that was sent was tabulated. For Prolaris^TM^, this included the genetic testing score, the 10-year risk of disease-specific mortality (DSM), the 10-year risk of metastasis disease, and the recommended treatment plan (single modality treatment or AS), calculated based off the Prolaris score and patient clinical factors [23].

Information on the timing of the Prolaris^TM^ sample was collected. We recorded whether the Prolaris^TM^ test was performed on a diagnostic biopsy or subsequent biopsy core. For subsequent biopsies, information on whether or not there was an upstaging of pathology from the diagnostic biopsy was also collected.

IBM SPSS v29.0 was used for statistical analyses. The demographic information of age and PSA was analyzed. We first compared the selective Prolaris^TM^ results of positive target cores versus positive systematic cores overall. Specifically, Chi-square and independent student’s *t* tests were utilized and an Alpha of 0.05 was used as the benchmark for significance. Where appropriate, Kruskal–Wallis and Mann–Whitney non-parametric testing was performed. Sub-analyses comparing systematic GG2 cancer versus MRI-target GG1 cancer, MRI-target GG1 versus non-target GG1, and GG2 MRI-target versus systematic GG2 were also performed. The analyses were re-performed looking specifically at the diagnostic biopsy cores and subsequent biopsy cores.

## 3. Results

A total of 264 patients met our inclusion criteria, 86 of whom had a Prolaris^TM^ test sent from a MRI-target cancer core and 178 whose test was sent from a systematic cancer core (Table 1). There was no statistically significant difference between these two groups in terms of age, PSA, or PSA density. Overall, 77.9% of the MRI-target cancers were GG1 on pathology while 22.1% were GG2. In the systematic biopsy cohort 64.6% of patients were GG1, while 35.4% were GG2. There was no statistically significant difference between the two groups in terms of the Prolaris^TM^ score, 10-year DSM, 10-year metastasis, or recommended treatment modality.

In our analysis of overall MRI-target GG1 versus and GG2 systematic cancer, we identified 67 GG1 patients and 63 GG2 patients (Table 2). There was no significant difference between the two groups for age, PSA, or PSA density. However, the GG2 systematic cancers were found to have significantly higher Prolaris^TM^ scores (3.4 vs. 3.18, *p* = 0.023), Prolaris^TM^-predicted 10-year DSM (3.4% vs. 2.3%, *p* < 0.01), and Prolaris^TM^ 10-year metastatic risk (1.9% vs. 1.2%, *p* = 0.013). This higher genomic risk resulted in a more aggressive management recommendation for GG2 non-target lesions; single-modal treatment was advised for 42.3% of GG2 systematic cancers and just 17.9% of GG1 MRI-target cancers (*p* < 0.01).

Additional analyses were conducted to compare GG1 MRI-target versus GG1 systematic cancers and GG2 MRI-target versus GG2 systematic cancers (Table 3). Both groupings had similar age, PSA, and PSA densities. In the GG1 analysis, there were 67 MRI-target cancers and 115 systematic cancers. MRI-target cancers were found to have a significantly higher Prolaris^TM^ score (3.18 vs. 2.99, *p* = 0.03), Prolaris^TM^-predicted 10-year DSM (2.35% vs. 2.05%, *p* = 0.034), and Prolaris^TM^-predicted 10-year metastatic risk (1.18% vs. 0.87%, *p* = 0.037) than their non-target counterparts. Nevertheless, there was no significant difference in treatment modality recommendation overall (17.9% vs. 12.2%, *p* = 0.29). In the GG2 MRI-target versus systematic cancer comparison, there were no statistically significant differences between the Prolaris^TM^ score, Prolaris^TM^-predicted 10-year DSM, and Prolaris^TM^-predicted 10-year metastatic risk. The treatment modality recommended by Prolaris^TM^ was not different for GG2 cancers.

A similar analysis was performed for diagnostic biopsies (Table 4, Table 5 and Table 6). There was no statistically significant difference in terms of age, PSA, or PSA density. There were 57 target cancers and 125 systematic cancers. There was no statistically significant difference between the two groups in terms of the Prolaris^TM^ score, 10-year DSM, 10-year metastasis, or recommended treatment modality.

In our analysis of diagnostic MRI-target GG1 versus GG2 systematic cancer, we identified 51 target GG1 patients and 41 GG2 patients. There were no significant differences for age, PSA, or PSA density. The GG2 systematic cancers were found to have a significantly higher Prolaris^TM^-predicted 10-year DSM (3.41% vs. 2.39%, *p* < 0.01), and Prolaris^TM^ 10-year metastatic risk (1.71% vs. 0.98%, *p* = 0.013). Although the systematic GG2 cancers had a higher risk score (3.42 vs. 3.17), this was not significant (*p* = 0.07). The test recommended a more aggressive management recommendation; single-modal treatment was advised for 53.7% of GG2 systematic cancers and just 19.6% of GG1 MRI-target cancers (*p* < 0.01).

Additional sub-analyses were conducted to compare diagnostic GG1 MRI-target versus GG1. Both groupings had similar age, PSA, and PSA densities. In the GG1 analysis, there were 51 MRI-target cancers and 84 systematic cancers. MRI-target cancers did not have a significantly higher Prolaris^TM^ score, Prolaris^TM^-predicted 10-year DSM, or Prolaris^TM^-predicted 10-year metastatic risk. There was no significant difference in treatment modality recommendation, although 19.6% of targets versus 14.2% of systematics were recommended treatment. There were only six target GG2 cancers in the diagnostic biopsy cohort.

Another analysis was performed for subsequent biopsies (Table 7, Table 8 and Table 9). There was no statistically significant difference in terms of age, PSA, or PSA density. There were 29 target cancers and 52 systematic cancers. There was no statistically significant difference between the two groups in terms of the Prolaris^TM^ score, 10-year DSM, 10-year metastasis, or recommended treatment modality.

In our analysis of subsequent MRI-target GG1 versus GG2 systematic cancer, we identified 16 target GG1 patients and 21 GG2 patients. Of the GG2 patients, 19 were the result of upstaging from GG1. There were no significant differences for age, PSA, or PSA density. The GG2 systematic cancers were found to have a significantly higher Prolaris^TM^-predicted 10-year DSM (3.0% vs. 2.2%, *p* = 0.02), and Prolaris^TM^ 10-year metastatic risk (1.31% vs. 0.6%, *p* = 0.01). Although lower, there was no significant difference (*p* = 0.25) in the treatment recommendation; 12.5% of MRI-target GG1 and 33% of systematic GG2 cancers were recommended single modal therapy.

Of the 19 patients who had disease upgrading, 5 also had initial prostate biopsy Prolaris^TM^ score reports. The initial and subsequent Prolaris^TM^ score mean (range) were 2.9 (2.1–3.2) and 3.3 (2.8–3.3), respectively. The DSM was 1.66% (1.0–2.2%) and 3.18% (2.4–4.6%). The metastasis risk was 0.7% (0.4–0.9%) and 1.6% (1.1–3.1%). The number of patients with recommended treatment of single modal therapy was 0 and then 2.

Additional sub-analyses were conducted to compare diagnostic GG1 MRI-target versus GG1 and GG2 MRI-target versus GG2 systematic cancers. Both groupings had similar age, PSA, and PSA densities. In the GG1 analysis, there were 16 MRI-target cancers and 31 systematic cancers. MRI-target cancers did not have a significantly higher Prolaris^TM^ score, Prolaris^TM^-predicted 10-year DSM, or Prolaris^TM^-predicted 10-year metastatic risk. There was no significant difference in treatment modality recommendation, although 12.5% targets versus 6.4% were recommended treatment. There were 13 target GG2 cancers. MRI-target cancers did not have a significantly higher Prolaris^TM^ score, Prolaris^TM^-predicted 10-year DSM, or Prolaris^TM^-predicted 10-year metastatic risk. There was no significant difference in treatment modality recommendation, although 61.5% targets versus 33% were recommended treatment.

## 4. Discussion

In our Prolaris^TM^ analysis, we found that MRI-visible cancer was not consistently associated with higher genetic testing risk scores in patients being considered for AS. When comparing overall GG1 MRI-target cancers to systematic cancers Prolaris^TM^ score, predicted 10-year DSM, and 10-year metastatic risk were significantly higher, but this did not significantly alter the recommended treatment course. However, when comparing MRI-target GG2 cancers versus systematic cancers, there were no significantly different scores or treatment recommendations. And when comparing MRI-target GG1 versus systematic GG2, the systematic GG2 cancers had a higher Prolaris^TM^ score, predicted 10-year DSM, and 10-year metastatic risk, as well as more aggressive treatment recommendations.

When analyzing the Prolaris ^TM^ on the diagnostic biopsy group, MRI-visible cancer was not associated with higher risk scores in any tests. We noted that again, when comparing MRI-target GG1 versus systematic GG2, the systematic GG2 cancers had a higher Prolaris ^TM^ risk profile and more aggressive treatment recommendation.

Interestingly, in analyzing Prolaris ^TM^ testing on our subsequent biopsy group, systematic GG2 had certain higher Prolaris ^TM^ risk factors, but were not significantly recommended more aggressive treatment modalities compared to target GG1 cancers.

A systematic review found that MRI-visible prostate cancers have more aggressive genetic features of tumor development, including proliferative signaling, DNA damage, and inflammatory processes [15]. A retrospective study compared Prolaris^TM^ scores with patients who had MRI within 6 months of prostate biopsy. They found that extracapsular extension was correlated with higher scores but not features such as MRI-visibility [24]. However, the study did not directly compare the risk profiles MRI-target cancers with systematic cancers. Our study is the first to analyze MRI-target prostate biopsy cores to systematic cores to help guide decision making for active surveillance with the goal of avoiding the morbidities of radical prostate cancer treatment.

Approximately 60% of low risk prostate cancer patients elect for AS protocol to manage their prostate cancer, with numbers continuing to rise [3]. Certain factors, such as GG2 and MRI-target cancer positivity place patients at risk of cancer upgrading [25]. Other factors, such as MRI-invisible GG1 prostate cancer only through systematic biopsy, place patients at lower risk and raise questions about the need for confirmatory prostate biopsy [26]. As a result, the evaluation of each individual patient for AS candidacy is paramount. Nonetheless, 10–15% patients on AS will experience tumor progression [22]. The use of multi-genetic testing for subsequent biopsies during AS is limited and there is minimal guidance provided by the NCCN and AUA. The use of Prolaris^TM^, which we demonstrate is not directly associated with MRI-visibility, may help guide the decision process, even in the confirmatory biopsy process. Specifically, in our study we demonstrate that GG2 on a subsequent biopsy appears to have a lower risk profile and treatment recommendation. Furthermore, the test still aids in clinical decision making, as it recommended radical treatment for 17.9% of GG1 target lesions and 12.2% of GG1 non-target lesions in our study population, a subset of patients who otherwise would likely been enrolled in active surveillance. In addition, 52.6% of target GG2 and 57.90% of non-target GG2 tumors were recommended to pursue AS, a significant number of patients who likely would have undergone overtreatment with radical therapy. A study demonstrated that management decision changed 65% of the time with a reduction in the invasiveness of therapy in 40% and an escalation in 24.9% of cases, which our findings corroborate [27].

Our study has several limitations, most importantly its retrospective and single-institution nature. By including consecutive patients undergoing mpMRI, biopsy, and genetic testing as well as only patients that are candidates for starting and continuing active surveillance, being the protocol at our institution, we aimed to minimize selection bias. Nevertheless, our study adds to the much-needed literature regarding the use of mpMRI and multi-genetic testing in prostate cancer diagnosis and longitudinal active surveillance.

## 5. Conclusions

Prolaris^TM^ testing for MRI-target cancers versus systematic cancers overall have similar scores, risk scores, and treatment recommendations. There is utility in performing Prolaris testing beyond the initial diagnostic biopsy, especially to assist in deciding whether to initiate or continue AS. Each individual case of prostate cancer and its corresponding genomic score report must be assessed to determine AS candidacy.

## Figures and Tables

**Table 1:** Prolaris^TM^—overall.

Variable	Target Lesion (n = 86)	Systematic Biopsy (n = 178)	*p*-Value
Age (years)	65.7 ± 10.7	65.5 ± 7.7	0.45
PSA (ng/mL)	6.8 ± 3.5	6.7 ± 3.8	0.84
PSA Density (ng/mL/cm^3^)	0.16 ± 0.13	0.16 ± 0.12	0.81
Gleason Grade Group			
-Grade 1 (%)	67 (77.90%)	115 (64.60%)	
-Grade 2 (%)	19 (22.10%)	63 (35.40%)	
Prolaris^TM^ Score (Mean ± SD)	3.23 ± 0.62	3.14 ± 0.72	0.18
Prolaris^TM^ 10-y DSM (Mean ± SD)	2.57% ± 1.37	2.54% ± 1.35	0.42
Prolaris^TM^ 10-y Metastasis (Mean ± SD)	1.44% ± 1.38	1.29% ± 1.13	0.21
Recommended Treatment			
-Active Surveillance (AS) (%)	75.60%	77.50%	0.74
-Single Modal Treatment (%)	24.40%	22.50%	

**Table 2:** Overall GG2 non-target versus GG1 target.

Variable	GG1 Target (n = 67)	GG2 Non-Target (n = 63)	*p*-Value
Age (years)	64.7 ± 11.1	67.7 ± 8.1	0.083
PSA (ng/mL)	6.9 ± 3.7	6.5 ± 2.7	0.44
PSA Density (ng/mL/cm^3^)	0.16 ± 0.14	0.17 ± 0.13	0.85
Prolaris^TM^ Score (Mean ± SD)	3.18 ± 0.66	3.4 ± 0.71	0.023
Prolaris^TM^ 10-y DSM (Mean ± SD)	2.3% ± 1.3	3.4% ± 1.6	<0.01
Prolaris^TM^ 10-y Metastasis (Mean ± SD)	1.2% ± 1.3	1.9% ± 1.4	0.013
Recommended Treatment			
-Active Surveillance (AS) (%)	82.10%	57.90%	<0.01
-Single Modal Treatment (%)	17.90%	42.10%	

**Table 3:** Overall MRI-target GG1 vs. GG1, and GG2 vs. GG2.

Variable	GG1 Target (n = 67)	GG1 Non-Target (n = 115)	*p*-Value
Age (years)	64.7 ± 11.1	65.9 ± 7.5	0.38
PSA (ng/mL)	6.9 ± 3.7	6.9 ± 4.3	0.91
PSA Density (ng/mL/cm^3^)	0.16 ± 0.14	0.15 ± 0.12	0.47
Prolaris^TM^ Score (Mean ± SD)	3.18 ± 0.66	2.99 ± 0.68	0.03
Prolaris^TM^ 10-y DSM (Mean ± SD)	2.35% ± 1.3	2.05% ± 0.85	0.034
Prolaris^TM^ 10-y Metastasis (Mean ± SD)	1.18%± 1.27	0.87% ± 0.58	0.037
Recommended Treatment			
-Active Surveillance (AS) (%)	82.10%	87.80%	0.29
-Single Modal Treatment (%)	17.90%	12.20%	
MRI-target GG2 vs. GG2			
**Variable**	**GG2 Target** **(n = 19)**	**GG2 Non-Target** **(n = 63)**	***p*** **-Value**
Age (years)	69.1 ± 8.4	67.7 ± 8.1	1.52
PSA (ng/mL)	6.4 ± 2.6	6.5 ± 2.7	0.93
PSA Density (ng/mL/cm^3^)	0.15 ± 0.1	0.17 ± 0.13	0.53
Prolaris^TM^ Score (Mean ± SD)	3.38 ± 0.43	3.42 ± 0.71	0.4
Prolaris^TM^ 10-y DSM (Mean ± SD)	3.38% ± 1.40	3.39% ± 1.61	0.49
Prolaris^TM^ 10-y Metastasis (Mean ± SD)	2.33% ± 1.38	1.86%± 1.42	0.14
Recommended Treatment			
-Active Surveillance (AS) (%)	52.60%	57.90%	0.79
-Single Modal Treatment (%)	47.40%	42.10%	

**Table 4:** Prolaris^TM^—diagnostic Biopsy.

Variable	Target Lesion (n = 57)	Systematic Biopsy (n = 125)	*p*-Value
Age (years)	65.5 ± 12.0	66.5 ± 8.1	0.49
PSA (ng/mL)	6.9 ± 3.7	6.9 ± 3.7	1
PSA Density (ng/mL/cm^3^)	0.16 ± 0.14	0.1 ± 0.14	0.99
Gleason Grade Group			
-Grade 1 (%)	51 (89.5%)	84 (67.2%)	
-Grade 2 (%)	6 (10.5%)	41 (33.8%)	
Prolaris^TM^ Score (Mean ± SD)	3.20 ± 0.64	3.20 ± 0.71	0.66
Prolaris^TM^ 10-y DSM (Mean ± SD)	2.49% ± 1.40	2.52% ± 1.29	0.89
Prolaris^TM^ 10-y Metastasis (Mean ± SD)	1.08% ± 1.34	0.98% ± 1.10	0.59
Recommended Treatment			
-Active Surveillance (AS) (%)	75.4%	75.2%	0.8
-Single Modal Treatment (%)	24.6%	24.8%	

**Table 5:** Diagnostic biopsy GG2 non-target versus GG1 target.

Variable	GG1 Target (n = 51)	GG2 Systematic (n = 41)	*p*-Value
Age (years)	64.7 ± 12.3	67.7 ± 8.7	0.19
PSA (ng/mL)	7.0 ± 3.8	7.1 ± 2.9	0.94
PSA Density (ng/mL/cm^3^)	0.17 ± 0.14	0.16 ± 0.16	0.56
Prolaris^TM^ Score (Mean ± SD)	3.17 ± 0.66	3.42 ± 0.70	0.073
Prolaris^TM^ 10-y DSM (Mean ± SD)	2.39% ± 1.4	3.41% ± 1.47	<0.01
Prolaris^TM^ 10-y Metastasis (Mean ± SD)	0.98% ± 1.36	1.71% ± 1	0.02
Recommended Treatment			
-Active Surveillance (AS) (%)	81.4%	53.7%	<0.01
-Single Modal Treatment (%)	19.6%	46.3%	

**Table 6:** Diagnostic biopsy MRI-target GG1 vs. systematic GG1.

Variable	GG1 Target (n = 51)	GG1 Non-Target (n = 84)	*p*-Value
Age (years)	64.7 ± 12.3	65.9 ± 7.8	0.5
PSA (ng/mL)	7.0 ± 3.0	6.8 ± 4.1	0.8
PSA Density (ng/mL/cm^3^)	0.17 ± 0.14	0.16 ± 0.13	0.66
Prolaris^TM^ Score (Mean ± SD)	3.20 ± 0.66	3.0 ± 0.69	0.21
Prolaris^TM^ 10-y DSM (Mean ± SD)	2.39% ± 1.4	2.08 ± 0.92	0.12
Prolaris^TM^ 10-y Metastasis (Mean ± SD)	0.98 ± 1.36	0.63 ± 0.66	0.06
Recommended Treatment			
-Active Surveillance (AS) (%)	80.4%	85.7%	0.74
-Single Modal Treatment (%)	19.6%	14.3%	

**Table 7:** Prolaris^TM^—subsequent biopsy.

Variable	Target Lesion (n = 29)	Systematic Biopsy (n = 52)	*p*-Value
Age (years)	66.0 ± 7.8	66.5 ± 6.8	0.78
PSA (ng/mL)	6.6 ± 3.1	6.3 ± 4.0	0.78
PSA Density (ng/mL/cm^3^)	0.15 ± 0.11	0.13 ± 0.06	0.24
Gleason Grade Group			
-Grade 1 (%)	16 (55.2)	31 (59.6)	
-Grade 2 (%)	13 (44.8)	21 (40.4)	
Prolaris^TM^ Score (Mean ± SD)	3.3 ± 0.6	3.1 ± 0.7	0.19
Prolaris^TM^ 10-y DSM (Mean ± SD)	2.7% ± 1.3	2.4% ± 0.99	0.23
Prolaris^TM^ 10-y Metastasis (Mean ± SD)	1.14 ± 1.4	0.81 ± 0.86	0.21
Recommended Treatment			
-Active Surveillance (AS) (%)	75.9%	82.7%	0.56
-Single Modal Treatment (%)	24.1%	17.3%	

**Table 8:** Subsequent biopsy GG2 non-target versus GG1 target.

Variable	GG1 Target (n = 67)	GG2 Non-Target (n = 63)	*p*-Value
Age (years)	64.6 ± 6.6	67.4 ± 6.9	0.23
PSA (ng/mL)	6.6 ± 3.3	5.4 ± 1.9	0.19
PSA Density (ng/mL/cm^3^)	0.15 ± 0.11	0.13 ± 0.05	0.65
Prolaris^TM^ Score (Mean ± SD)	3.2% ± 0.66	3.3% ± 0.63	0.64
Prolaris^TM^ 10-y DSM (Mean ± SD)	2.2% ± 0.8	3.0% ± 1.1	0.02
Prolaris^TM^ 10-y Metastasis (Mean ± SD)	0.6% ± 0.5	1.31% ± 1.0	0.01
Recommended Treatment			
-Active Surveillance (AS) (%)	87.5%	67.7%	0.25
-Single Modal Treatment (%)	12.5%	33.3%	

**Table 9:** Subsequent biopsy MRI-target GG1 vs. GG1 systematic and MRI-target GG2 vs. systematic GG2.

Variable	GG1 Target (n = 16)	GG1 Non-Target (n = 31)	*p*-Value
Age (years)	64.6 ± 6.6	65.9 ± 6.7	0.53
PSA (ng/mL)	6.6 ± 3.3	6.9 ± 5.0	0.83
PSA Density (ng/mL/cm^3^)	0.15 ± 0.11	0.12 ± 0.07	0.41
Prolaris^TM^ Score (Mean ± SD)	3.2 ± 0.66	2.99 ± 0.68	0.13
Prolaris^TM^ 10-y DSM (Mean ± SD)	2.35% ± 1.3	2.9% ± 0.7	0.33
Prolaris^TM^ 10-y Metastasis (Mean± SD)	2.2% ± 0.8	1.98% ± 0.65	0.28
Recommended Treatment			
-Active Surveillance (AS) (%)	87.5%	93.1%	0.6
-Single Modal Treatment (%)	12.5%	6.9%	
	**GG2 Target** **(n = 13)**	**GG2 Non-Target** **(n = 21)**	***p*** **-Value**
Age (years)	67.8 ± 9.1	6.4 ± 6.9	0.88
PSA (ng/mL)	6.6 ± 2.9	5.4 ± 1.9	0.15
PSA Density (ng/mL/cm^3^)	0.16 ± 0.11	0.13 ± 0.05	0.41
Prolaris^TM^ Score (Mean ± SD)	3.3 ± 0.5	3.3 ± 0.6	0.98
Prolaris^TM^ 10-y DSM (Mean ± SD)	3.4% ± 1.5	3.01% ± 1.1	0.43
Prolaris^TM^ 10-y Metastasis (Mean ± SD)	1.77% ± 1.85	1.31% ± 1.1	0.37
Recommended Treatment			
-Active Surveillance (AS) (%)	39.5%	67.7%	1
-Single Modal Treatment (%)	61.5%	33.3%	

## Data Availability

The data presented in this study are available on request from the corresponding author.
